# Pan-cancer analysis of somatic mutations and epigenetic alterations in insulated neighbourhood boundaries

**DOI:** 10.1371/journal.pone.0227180

**Published:** 2020-01-16

**Authors:** Pietro Pinoli, Eirini Stamoulakatou, An-Phi Nguyen, María Rodríguez Martínez, Stefano Ceri

**Affiliations:** 1 DEIB, Politecnico di Milano, Milano, Italy; 2 IBM Research Zürich, Rüschlikon, Switzerland; McGill University, CANADA

## Abstract

Recent evidence shows that the disruption of constitutive insulated neighbourhoods might lead to oncogene dysregulation. We present here a systematic pan-cancer characterisation of the associations between constitutive boundaries and genome alterations in cancer. Specifically, we investigate the enrichment of somatic mutation, abnormal methylation, and copy number alteration events in the proximity of CTCF bindings overlapping with topological boundaries (junctions) in 26 cancer types. Focusing on CTCF motifs that are both *in-boundary* (overlapping with junctions) and *active* (overlapping with peaks of CTCF expression), we find a significant enrichment of somatic mutations in several cancer types. Furthermore, mutated junctions are significantly conserved across cancer types, and we also observe a positive selection of transversions rather than transitions in many cancer types. We also analyzed the mutational signature found on the different classes of CTCF motifs, finding some signatures (such as SBS26) to have a higher weight within in-boundary than off-bounday motifs. Regarding methylation, we find a significant number of over-methylated active in-boundary CTCF motifs in several cancer types; similarly to somatic-mutated junctions, they also have a significant conservation across cancer types. Finally, in several cancer types we observe that copy number alterations tend to overlap with active junctions more often than in matched normal samples. While several articles have recently reported a mutational enrichment at CTCF binding sites for specific cancer types, our analysis is pan-cancer and investigates abnormal methylation and copy number alterations in addition to somatic mutations. Our method is fully replicable and suggests several follow-up tumour-specific analyses.

## Introduction

The human genome is organized hierarchically into discrete topologically associated domains (TADs), sub-megabase segments that tend to self-associate and are relatively insulated from neighboring domains [1, 2]. TAD boundaries, revealed by high-throughput chromatin conformation capture (HiC) techniques [3], are enriched for the insulator binding protein CTCF, which plays a central role in multiple complex genomic processes, including transcription [4, 5], imprinting [6, 7], long-range chromatin interactions and subnuclear localization [8, 9].

TADs are hierarchical in nature [10, 11] and usually contain smaller sub-TADs and individual loops that function as insulated neighborhoods [12, 13]. While TADs are largely invariant features of genome organization and are mostly conserved across cell types and even across species, sub-TADs, loops, and insulated neighborhoods appear to differ, at least partially, between different cell lineages [14].

Loss of CTCF-mediated insulation has been identified as a potential reason for domain disruption and spreading [15] and has been connected with multiple malignancies, such as cancer [16–18], intellectual disability [19, 20] and developmental disorders [21]. In cancer, recent studies have shown that TAD disruption is often found in cancer cells and contributes to oncogenesis through several mechanisms. For instance, mutated or epigenetic alterations of a TAD boundary might lead to the fusion of two adjacent TADs [13, 18, 22, 23]. Hypermethylation of CTCF binding sites has been observed to lead to loss of insulation between topological domains and consequent aberrant gene activation in gliomas [22]. Microdeletions that eliminate the boundary sites of insulated neighborhoods containing prominent acute lymphoblastic leukemia proto-oncogenes have also been reported [23]. The same study also identified an enrichment in boundary CTCF site mutations in the genomes of esophageal and liver carcinoma [23]. Furthermore, genomic rearrangements with breakpoints within TADs can lead to breakage or fusion of TADs that might result in oncogene activation [24–27], as observed in prostate cancer, where chromosomal deletions lead to the establishment of new domain boundaries and the rearrangement of gene interactions [27]. Hotspots of mutations within CTCF motifs have been independently observed in melanoma [28, 29] related to UV exposure, and in gastrointestinal cancers [30]. In parallel, statistical analyses have showed an enrichment of cancer-associated genes in proximity of mutated boundaries [28] and highlighted specific dysregulated genes [30]. Furthermore, 21 insulators (both cancer-specific and pan-cancer) have been suggested as cancer drivers in recent work [31]. Finally, a recent review [32] summarizes various findings about the increase of somatic mutations at binding sites of TFs including CTCF, and suggests that the mutation frequency increase is shaped by the complex interplay between DNA damage and repair levels. These studies have motivated our work to systematically characterize the dysregulation of CTCF binding across different cancer types and through different mechanisms of dysregulation.

## Materials and methods

We first outline our approach to detect insulated neighbourhoods and CTCF binding motifs. To investigate mutations that can potentially affect TADs, we analyzed public datasets available from the International Cancer Genome Consortium (ICGC [33]) and The Cancer Genome Atlas (TCGA [34]), leveraging data from the ENCODE Consortium [35]. Table A in S1 File lists the cancer types we have considered. For the systematic exploration of large datasets we used a novel approach for genomic computing [36].

Regarding the definition of insulated neighbourhoods, we considered three datasets that have mapped insulated neighbourhoods using Chromatin Interaction Analysis by Paired-End Tag (ChIA-PET):

1. A breast cancer study that characterised CTCF-mediated chromatin interactions using ChIA-PET experiments on the MCF7 breast cancer cell line [37].
2. A study on the H1-hESC human embryonic stem cell line that inferred neighbourhoods using the SMC1 protein—cohesin subunit as the target of the ChIA-PET experiment [13].
3. A set of constitutive neighbourhoods provided by Hnisz and colleagues [23]. This set was obtained as the intersection of two RAD21, a subunit of the cohesin complex, ChIA-PET experiments on GM12878 and K562 cell lines, and a SMC1 ChIA-PET experiment on a Jurkat cell line. Only neighbourhoods confirmed in at least 2 cell lines were kept.

**Fig 1:**
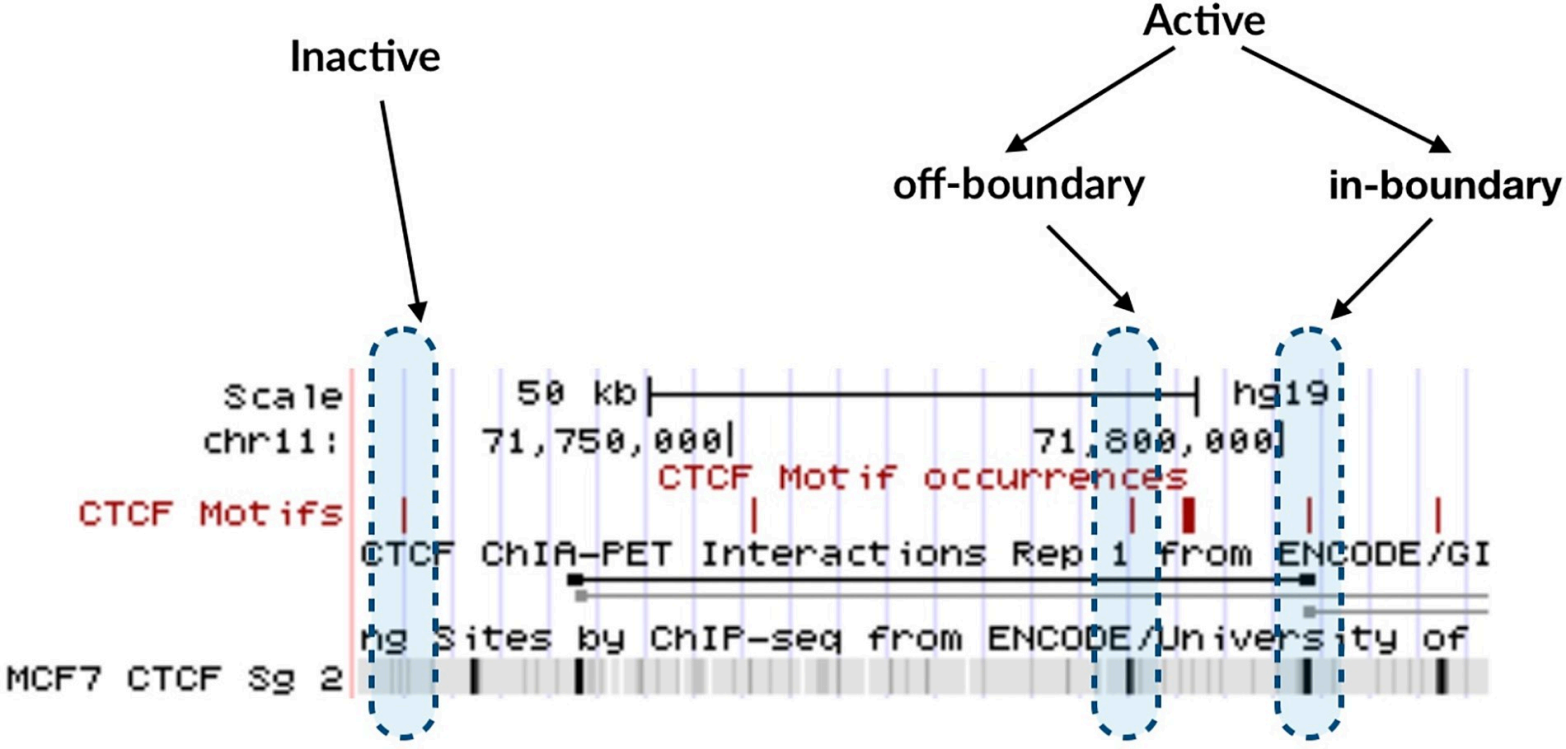
Classification of CTCF motifs, within a short portion of chromosome 11. Motifs are classified as active (confirmed by a CTCF ChIP-seq peak) and inactive (not confirmed). Active motifs are further divided into in-boundary and off-boundary according to whether they overlap a boundary, as defined by a ChIA-PET experiment.

We used Biostrings [38] to identify the CTCF binding sites. Specifically, we used the 19bp long Jaspar [39] MA0139.1 motif (Fig A in S1 File) in the HG19 assembly, finding 107,230 (104,459 excluding chromosome Y) positions matching the motif with a score of at least 80%. Next, we classified motifs as *in-boundary/off-boundary* and *active/inactive* according to whether they were located respectively on a previously mapped insulated neighbourhood and on a peak of CTCF ChIP-seq data, as characterised by the Bernstein Laboratory and published on ENCODE [35] (ENCSR000AMF for H1-hESC, ENCSR000AKB for GM12878 and ENCSR560BUE for MCF7). Fig 1 illustrates the different types of CTCF motifs according to the above classification, while the Table 1 reports the number of boundaries and motifs in each category for each ChIA-PET dataset and ChIP-seq cell line.

**Table 1:** Summary statistics of the number of boundaries and motifs. The first two experiments performed ChIA-PET experiments on an MCF7 breast cancer cell line [37] and an H1-hESC human embryonic stem cell line [13]. The Hnisz dataset is defined as the intersection of three ChIA-PET experiments targeting respectively RAD21 on cell lines GM128178 and K562, and SMC1 on a Jurkat cell line, with only neighbourhoods predicted in at least 2 out of 3 ChIA-PET experiments being considered.

ChIA-PET DataSet	ChIP-seq cellLine	Number of boundaries	Active in-bnd.	Active off-bnd.	Inactive in-bnd.
**MCF7**	MCF7	34,052	11,825	16,570	1,321
**hESC**	H1-hESC	47,274	11,907	6,929	2,113
**Hnisz**	GM12878	16,437	12,815	15,840	323

### Point mutation analysis

#### Enrichment of somatic mutations on active, in-boundary CTCF motifs

While mutations in CTCF binding sites have been reported to occur frequently in several cancers, the significance of these findings across the broad spectrum of cancer types has not yet been evaluated. In order to test the enrichment of mutations on CTCF sites, we analyzed Whole Genome Sequencing (WGS) data publicly available at ICGC [33]. Specifically, we selected 14 cancer types for which at least 200,000 mutations across all patients were reported—this threshold was imposed in order to ensure adequate statistics. Opposed to earlier approaches that focused on identifying motif-disrupting mutations [28], we opt for a descriptive unbiased approach and choose to summarise all mutations identified in the 19bp long CTCF motif. Table B in S1 File summarizes the list of analyzed datasets and also reports the number of samples and total number of somatic mutations per cancer type.

**Fig 2:**
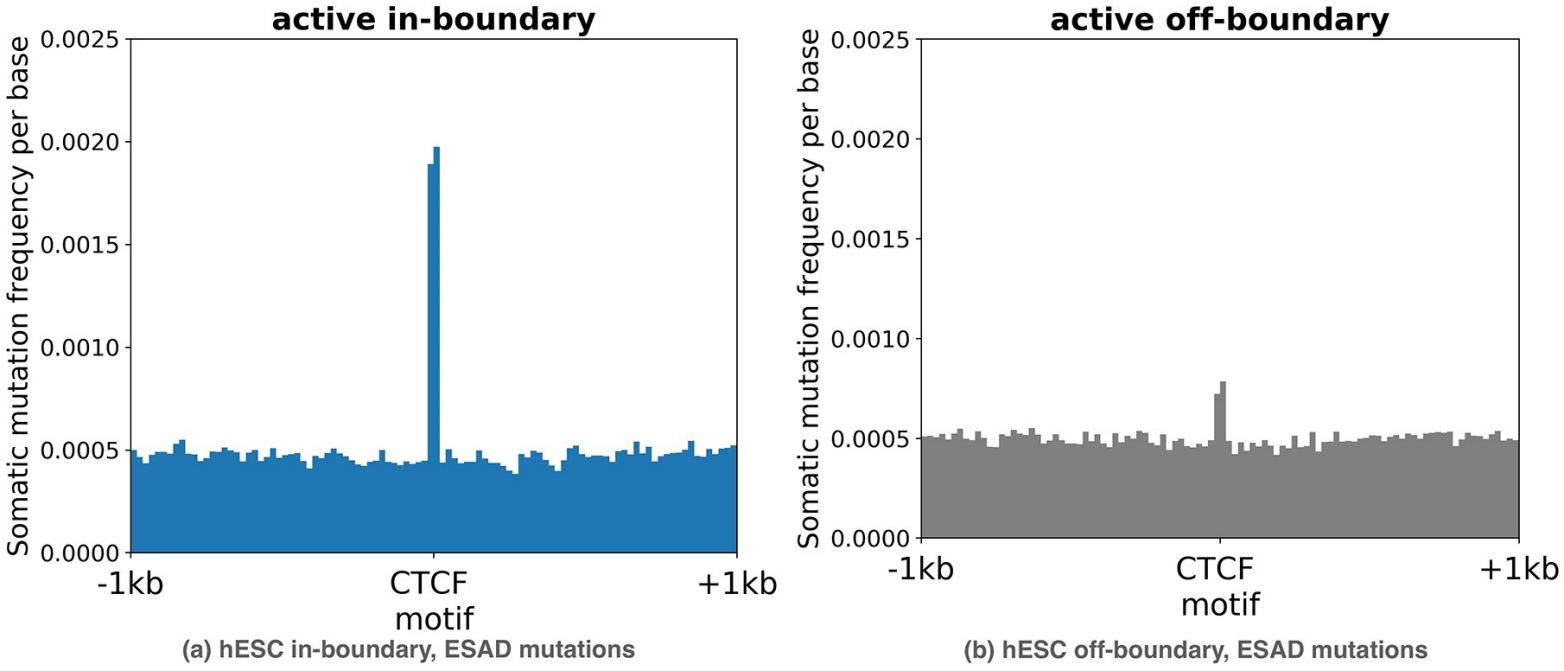
Somatic mutation frequency in a range of (-1kbp, +1kbp) surrounding (a) an active in-boundary and (b) an active off-boundary CTCF motifs in eEsophageal adenocarcinoma (ESAD). A central peak overlapping the 19bp CTCF motif is evident in (a).

Using GMQL [36], an in-house developed query language for genomic analyses, we observed an accumulation of somatic mutations on active in-boundary CTCF motifs. Fig 2, left subplot, shows the accumulation of mutations in esophageal adenocarcinoma (ESAD), one of the tumour types for which this phenomenon was more evident. As is evident in the figure, a peak in the mutation frequency distribution is clearly visible over the 19 bp CTCF active in-boundary motifs, while the mutation frequency over flanking regions is much smaller. In comparison, no such enrichment was found on active off-boundary motifs (Fig 2, right subplot). We find similar patterns in almost all other examined cancer types (see Fig B in S1 File for boundaries extracted from the ChIA-PET on the MCF7 cell line, Fig C in S1 File for boundaries from the hESC cell line, and Fig D in S1 File for the Hnisz dataset). This suggests that active, in-boundary CTCF motifs tend to acquire a larger number of mutations than expected by chance.

To estimate the statistical significance of the observed enrichment, we performed a per-patient analysis. Namely, for each patient we counted the number of mutations on in-boundary versus off-boundary motifs; we discarded patients with less than five mutations in at least on of the categories. We then created a two column contingency table, where the two columns correspond to in-boundary and off-boundary motifs, respectively, while each row corresponds to one of the considered patients. Finally, we run a chi-square test to test whether the distributions on the two columns are identical or distinct. The p-values of the chi-square test are reported in Table 2. We found the enrichment of somatic mutations in active in-boundary CTCF motifs to be significant in several cancer types and across the three datasets of insulated neighbourhoods.

**Table 2:** Significance of the enrichment of somatic mutations in active, in-boundary motifs for all studied cancer types and neighbourhoods datasets. The p-values are computed using a chi-square test. Within parenthesis are the number of patient on which each test have ben run. Cancer names are defined in Table A in S1 File, according to ICGC nomenclature.

tumour	hESC (# patients)	MCF7 (# patients)	Hnisz (# patients)
**ESAD**	0.0 (19)	4.22e-116 (65)	7.66e-191 (107)
**LIRI**	0.0 (4)	2.55e-60 (9)	1.79e-82 (12)
**BRCA**	0.41 (4)	1.05e-15 (7)	1.69e-08 (13)
**MELA**	0.0 (67)	6.43e-101 (110)	2.2e-241 (113)
**GACA**	0.373 (2)	2.77e-06 (6)	2.75e-07 (12)
**SKCA**	0.0 (17)	7.46e-05 (32)	2.72e-63 (33)
**MALY**	1.25 e-09 (5)	0.005 (8)	0.004 (9)
**COCA**	0.2 (3)	2.29e-08 (9)	0.003 (9)

Interestingly, in five cancer types, namely Esophageal Adenocarcinoma (ESAD), Liver Cancer (LIRI), Melanoma (MELA), Skin Adenocarcinoma (SKCA) and Malignant Lymphoma (MALY), the enrichment is significant in all the datasets of insulated neighbourhoods; in breast cancer, gastric cancer and colorectal cancer it is significant in MCF7 and *Hnisz* datasets. The *Hnisz* dataset is associated with the most significant results in most cancer types. We speculate that, since these neighbourhoods were obtained by intersecting different cell lines, they are more likely to be constitutive for the cell and, therefore, be present in most of the tissues.

#### Mutation enrichment in gene promoters and exons

Neighbourhood boundaries tend to fall in promoter regions where chromatin is open to facilitate transcription, especially in active genes, [40]. Hence, it could be argued that the enrichment of mutations in active in-boundary motifs is due to the proximity of these motifs to promoters, rather than to a cancer specific mechanism. To rule out this possibility, we divided the active CTCF motifs into two classes: motifs overlapping gene promoters and motifs outside promoter regions. Here, promoters were defined as the genomic regions spanning -2kb to +2kb from the transcription start site of a RefSeq annotated gene. We then computed the accumulation of mutations in these two classes of active motifs.

**Fig 3:**
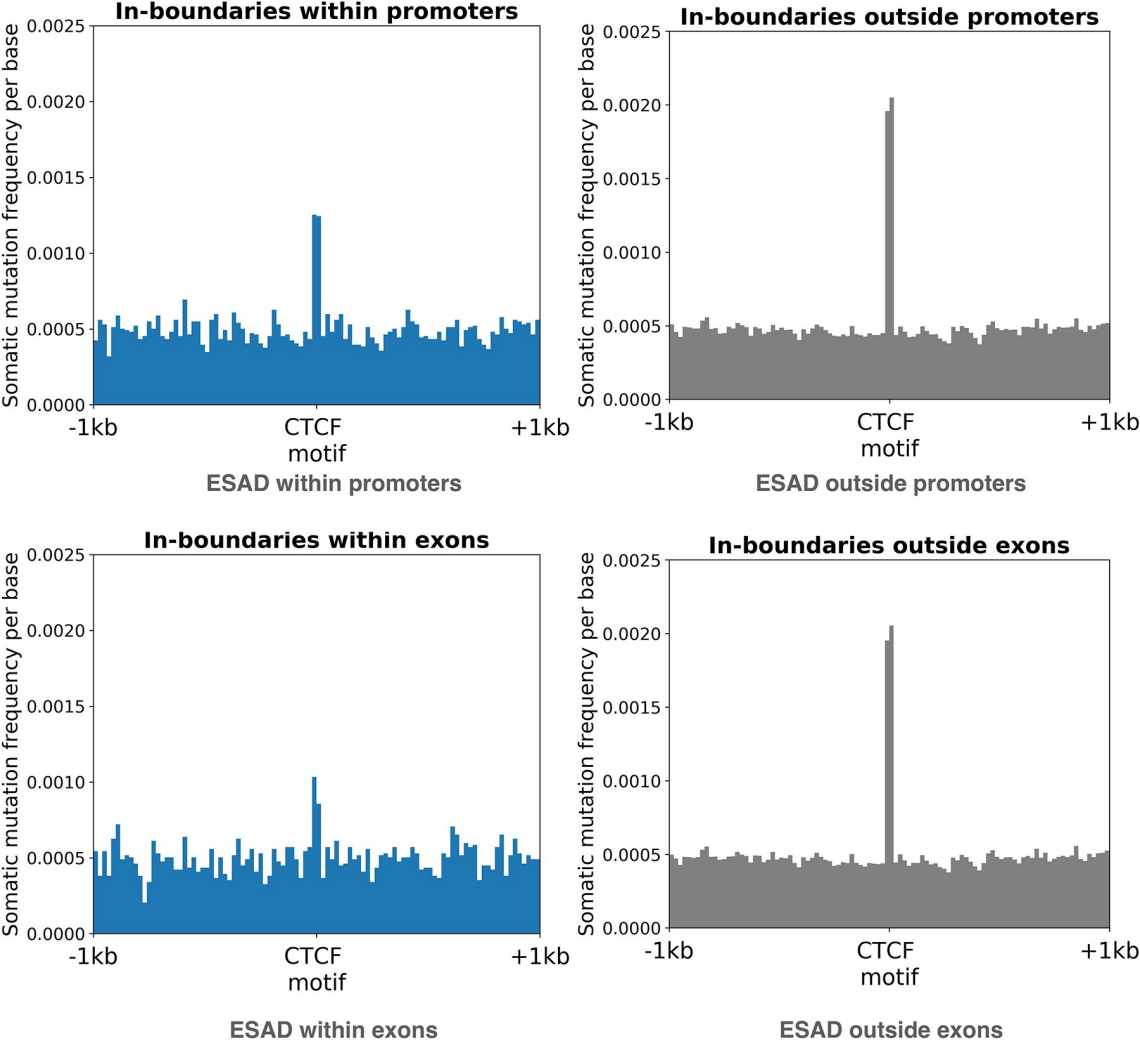
Distribution of the number of mutations on active in-boundary CTCF motifs within and outside promoters and exons in Esophageal Adenocarcinoma using the hESC cell line. A higher frequency of mutations is found outside promoters and exons, ruling out the possibility that the enrichment of somatic mutations in active CTCF motifs is due to their higher probability of having open chromatin.

Fig 3(a) and 3(b) shows the results of this analysis in Esophageal Adenocarcinoma (active CTCF motifs taken from the hESC dataset). A slightly higher frequency of somatic mutations is observable on active motifs outside promoters, ruling out the possibility that the enrichment in mutations is driven by the presence of a promoter. We performed similar analyses on all available cancer types (Table B in S1 File) and quantified the enrichment using the per-patient test, as described above. Empirical p-values are reported in the Table C in S1 File. In no cancer type did we observe an enrichment of mutations in promoters, supporting the hypothesis that the accumulation of mutations in active CTCF motifs is not due to the overlap of promoters, and hence, not due to the open state of the chromatin.

We also tested the difference of mutation enrichment for the boundaries colocated with the exons of protein coding genes. Fig 3(c) and 3(d) shows the results of this analysis in Esophageal Adenocarcinoma (active CTCF motifs taken from the hESC dataset). Again, a sligthly higher frequency of somatic mutations is observed in CTCF motifs outside exons, ruling out the hypothesis that such enrichment is due to the colocation with open chromatin regions.

We also tried to test if mutations tend to cluster on active enhancers, defined as the intersection of H3K4me1 and H3K27Ac histone modifications, but for such regions we found a very small intersection with active in-boundary CTCF motifs and it was not possible to perform a significative statistical test.

#### Genes close to frequently mutated boundaries

**Fig 4:**
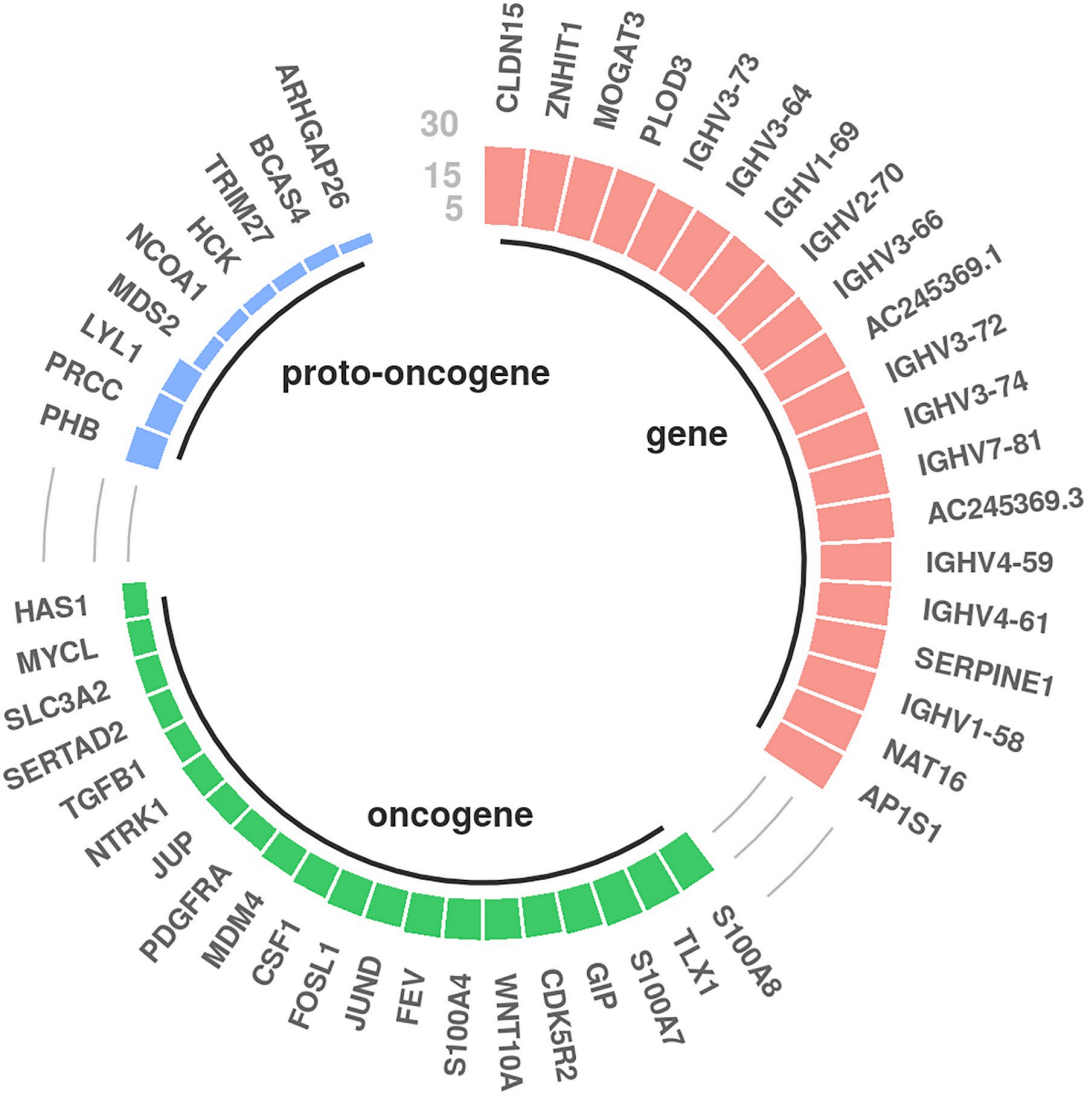
Genes close to mutated CTCF in-boundary motifs in melanoma. The height of the bars represents the number of samples in which the gene is close to a mutated in-boundary active CTCF motif. We considered only genes with top mutations whose TSS is within 180 kbp from the center of the mutated motif.

We also look for genes close to mutated in-boundary motifs. In Fig 4, we report the genes having a TSS within 180 kbp from a mutated active in-boundary motif for melanoma (180 kpb is the average size of an insulated neighbourhood). The plot shows that several oncogenes and proto-oncogenes are in the near neighbourhood of some mutated junctions. Notably, in the list of oncogenes we find TGFB1, whose up-regulation has been recently associated with disruption of CTCF binding motif due to somatic mutations in the melanoma A375 cell line [31]. We also find PDGFRA, an oncogene associated with an array of clinically significant neoplasms, also reported by Flavahan et al. [22] as dysregulated due to its proximity to a mutated boundary in gliomas. Circular plots for the other four cancer types with a significant mutational enrichment in all cell lines can be found in the Fig G in S1 File.

#### Analysis of the most frequent types of mutations

Next, we investigated whether the somatic mutations in active in-boundary motifs preferentially belong to a certain class of mutations, which could point to a specific tumourigenic mechanism. Hence, we split somatic point mutations into transitions (A↔G and C↔T) and transversions (A↔C, G↔T and A↔T, C↔G) and counted how many mutations fall into each category for each tumour. Table 3 reports the counts of transition and transversion mutations for active in-boundary and active off-boundary motifs for all considered cancers. We used a chi-square test on the contingency table associated with each cancer to assess whether the observed frequencies were homogeneously distributed between transitions and transversions. For seven cancer types—Esophageal Adenocarcinoma, Pancreatic Cancer Endocrine Neoplasm, Skin Cancer, Liver Cancer, Skin Adenocarcinoma, Malignant Lymphoma and Colorectal Cancer—one type of mutation occurred more frequently than expected by chance. Overall, most cancers acquire a similar or higher number of transversions than transitions, with the notable exception of skin cancers, where transitions are more frequent.

**Table 3:** Counts of transition and transversion mutations in in-boundary and off-boundary active motifs using the hESC dataset to define insulated neighbourhoods. P-values are computed using a chi-square test on the contingency table associated with each tumour.

	Class	Transitions	Transversions	p-value
**ESAD**	in-boundary	2858	3061	2.1e-12
	off-boundary	1461	1123	
**PACA**	in-boundary	260	300	5.10e-7
	off-boundary	212	119	
**MELA**	in-boundary	9173	628	7.2e-3
	off-boundary	5097	417	
**LIRI**	in-boundary	1117	1149	9.3e-3
	off-boundary	482	402	
**SKCA**	in-boundary	2567	409	0.029
	off-boundary	1458	280	
**MALY**	in-boundary	613	537	0.039
	off-boundary	436	313	
**COCA**	in-boundary	29	31	0.049
	off-boundary	35	16	
**BRCA**	in-boundary	1024	1112	0.058
	off-boundary	545	512	
**BTCA**	in-boundary	129	107	0.067
	off-boundary	76	40	
**OV**	in-boundary	298	406	0.089
	off-boundary	143	245	
**RECA**	in-boundary	141	197	0.150
	off-boundary	84	88	
**EOPC**	in-boundary	146	128	0.445
	off-boundary	82	60	
**BOCA**	in-boundary	58	51	0.893
	off-boundary	31	27	
**GACA**	in-boundary	120	118	0.949
	off-boundary	51	48	

In general, the ratio of transitions versus transversions depends on the type of DNA element. In particular, the ratio in binding sites and regulatory regions is not well characterized, although we note recent results in germline mutations [41] that indicate that transversions have a slightly larger effect on DNA shape and transcription factor binding than transitions. Assuming that similar results can be generalised to somatic alterations, this finding suggests that transversions on CTCF motifs might have a stronger effect than transitions, which could hint towards a positive selection of transversions rather than transitions in insulated neighborhood boundaries in tumour cells, as these alterations are more likely to disrupt boundaries than transitions.

**Fig 5:**
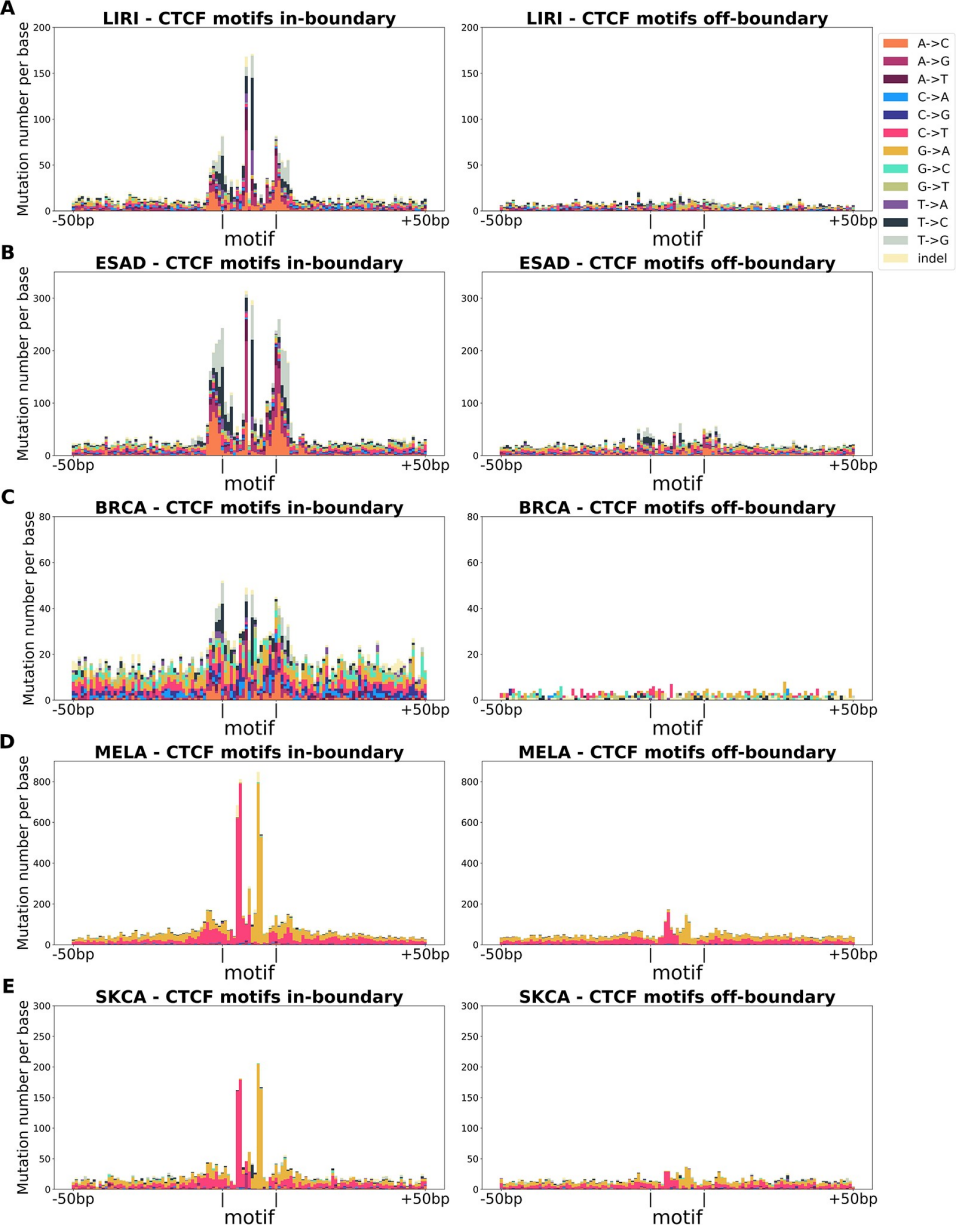
Mutations in active in-boundary CTCF motifs and flanking regions (19 bp ±50 bp). Clear peaks are observed within CTCF motifs in all cancers types. Additional peaks are observed in the flanking regions of the CTCF motifs in Liver Cancer, Esophageal Adenocarcinoma and Breast Cancer tumour types (sub-plots A,B,C).

Motivated by the non-random occurrences of transitions and tranversions in certain cancer types, we further investigated the mutation patterns most frequently found in the active CTCF motifs and flanking areas (19 bp ± 50 bp). We considered the five tumour types for which we have the highest number of mutations (see Table B in S1 File)—and studied their mutation patterns. Results are summarized in Fig 5.

Esophageal Adenocarcinoma (ESAD), Liver Cancer (LIRI) and Breast Cancer (BRCA) were associated with the largest enrichment of in-boundary mutations in all 3 considered ChIA-PET experiments (see Table 2). In Esophageal Adenocarcinoma and Liver Cancer, we note two very distinguishable peaks at positions 9 and 11 (the two positions flanking the central position of the motif, located at x—coordinate 10, see Fig 5A and 5B). Similar although less prominent peaks were also found in Breast Cancer (see Fig 5C). We further note significant peaks of mutations just outside the CTCF binding site, particularly marked in Esophageal Adenocarcinoma and Breast Cancer. We speculate that these mutations might correspond to the binding sites of the subunits of the largest cohesin complex that is assembled to define a boundary.

Point mutations in skin cancer (MELA) and skin adenocarcinoma (SKCA) are shown in Fig 5D and 5E. We note a prevalence of G→A mutations in positions 6-7 and C→T mutations in positions 13-14, which are symmetric with respect to the motif center at basis 10. These mutations are consistent with the observed enrichment of C→T and CC→TT mutations in ultraviolet exposure-driven melanoma tumours [42]. In these cases, no additional peaks in the flanking regions of the CTCF motif are observed.

#### Analysis of mutational signatures

**Fig 6:**
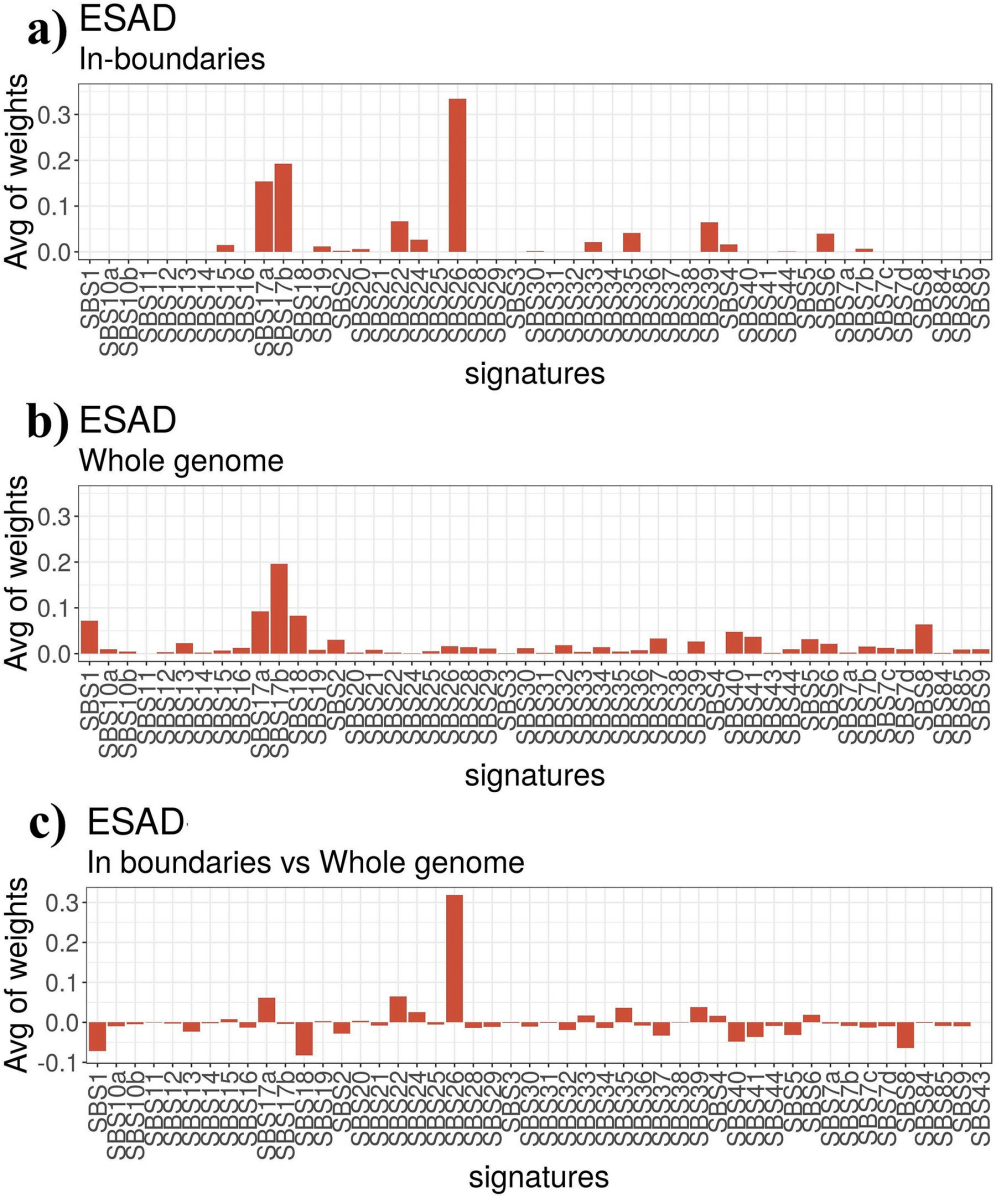
The average contributions or weights (i.e., exposures) of mutational signatures for the Esophageal Adenocarcinoma dataset. Signature refitting was done based on mutations falling a) in-boundaries motifs, b)in the whole genome, and and then c) on the difference between the two.

We next investigated whether the active in-boundary motifs on which we observed the mutation enrichment are affected by specific mutational processes. In order to do so, we computed the exposure (or weight contribution) of the Alexandrov mutation signatures [43] on in-boundary motifs and on the whole genome. An Alexandrov mutational signature describes a mutational process as a vector of mutation probabilities for all the 96 possible single nucleotide variants within their context of adjacent bases, i.e., each of these 96 probabilities corresponds to a triplet whose central base is mutated (e.g. G[G → A]A). We considered the catalogue of single base substitution mutational signatures provider by COSMIC and computed the relative exposure of each of them in the considered tumors; this operation, known as signature refitting, was performed by means of the Bioconductor R package decompTumor2Sig [44]. The set of mutational signatures found in a tumor (or in a particular class of genome regions) depends on which mutational processes were active and the related exposure indicates how strongly such process contributes to the mutational load in the tumor (or within the specific regions). In our analysis we found that signature SBS26, which is associated with defective DNA mismatch repair, is particularly prominent in the active in-boundary motifs in Esophageal Adenocarcinoma (ESAD Fig 6), Liver Cancer (LIRI, Fig I in S1 File) and Breast Cancer (BRCA, Fig H in S1 File), although the exposure of the same signature is not relevant in the whole genome of the same tumors. For what it concerns Skin Sancer (MELA) and Skin Adenocarcinoma (SKCA) we found that within in-boundary motifs the signature SBS7b has the highest exposure and not signature SBS7a as in the whole genome (Figs J and K in S1 File). Both signatures are associated with exposure to ultraviolet light and their most frequent mutation type is T[C → T]C (i.e., G[G→A]A) whose corresponding triplet TCC—GGA is present in a significant subset of CTCF binding sites (e.g., positions 3-5 and 13-15).

#### Overlap of mutated boundaries across cancer types

We also found a significant overlap of frequently mutated boundaries across tumour types in the same five tumour types (Melanoma, Esophageal Adenocarcinoma, Skin Adenocarcinoma, Liver Cancer and Breast Cancer) associated with the highest number of mutations, see Fig F in S1 File. We tested the significance of the pair-wise overlap of junctions across cancer types using the hypergeometric test. Results are shown in Table G in S1 File. All pair-wise comparisons resulted in very significant p-values, confirming that mutations in boundaries do not happen by random chance, hinting to a concerted oncogenic mechanism to dysregulate key cancer driver genes.

### Methylation analysis

The CTCF consensus binding sequence contains a CpG dinucleotide and can, therefore, be methylated—20% of CTCF binding sites are reported to be methylated on average [45]. Furthermore, it has been reported that up to 41% of variable CTCF binding is linked to differential DNA methylation, concentrated at the two CpG dinucleotides on the CTCF motif [46]. Increased methylation leading to disruption of CTCF binding patterns has also been observed in immortalized cell lines [46], suggesting that abnormal methylation of CTFC motifs might be a mechanism of cancer gene dysregulation. Hence, we decided to investigate if hyper-methylation occurs on in-boudary motifs, considering for this analysis 12 cancer types for which at least 20 matched tumoral and normal methylation samples are available in TCGA [34]. Table D in S1 File lists the available datasets. Active in boundary CTCF motifs are preferentially located on coding and regulatory elements, which indirectly reflects the distribution of methylation probes, preferentially located in coding and regulatory elements (see Table E in S1 File).

**Fig 7:**
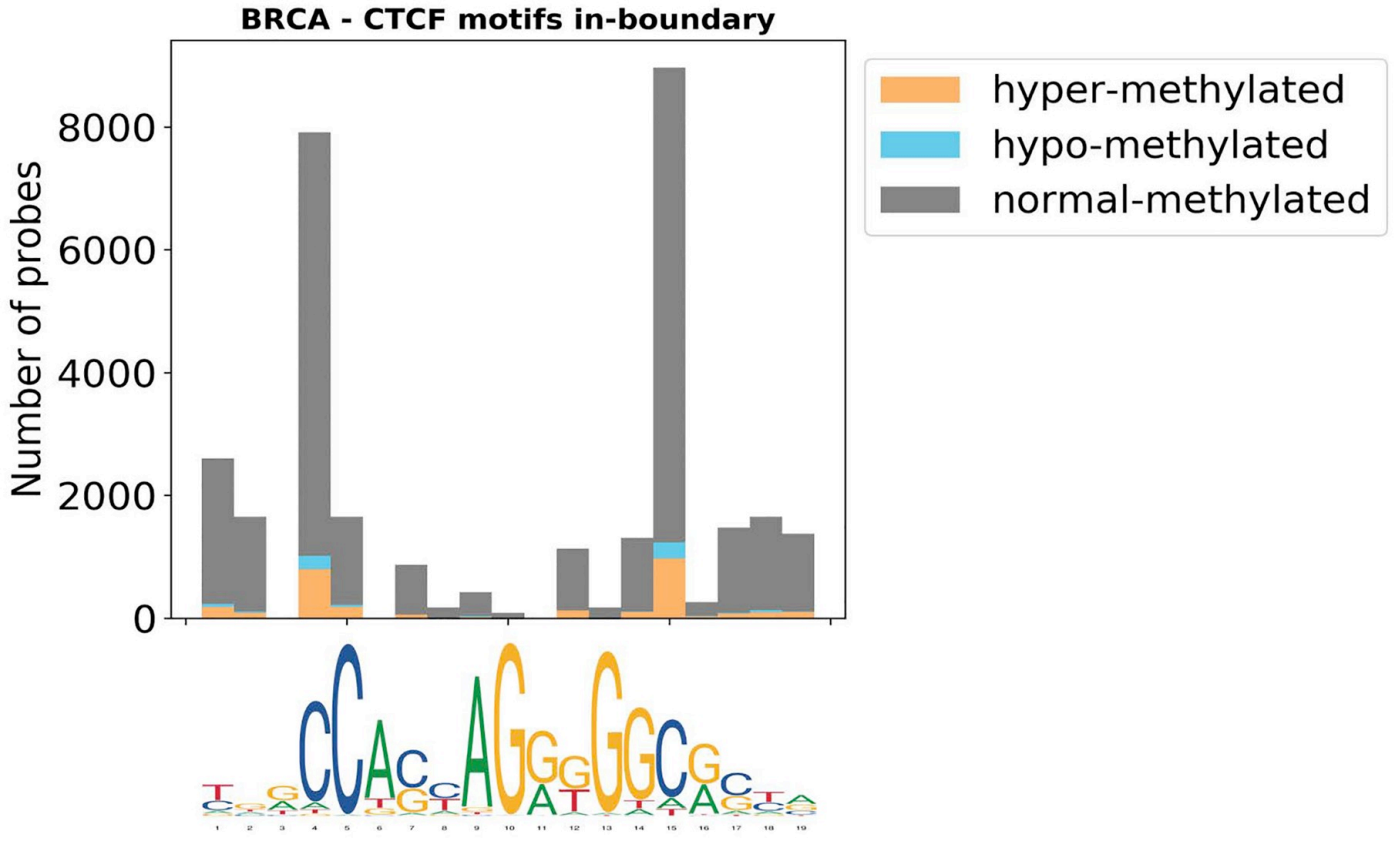
A representative distribution of delta methylation values on CTFC in-boundary motifs for Breast Cancer. Hypo-methylated refers to probes with a delta beta value lower than -0.2, hyper-methylated to probes with a delta beta value greater than 0.2 and normal-methylated to all the others. The x-axis reports the CTCF motif. Two distinguishable methylation peaks are observed at positions 4 and 15, corresponding to the presence of 2 pairs of CpG dinucleotides.

To investigate the effect of methylation on CTCF DNA binding, we first looked at the methylation patterns found on CTCF motifs. Fig 7 shows a representative example of the distribution of the delta of methylation intensities (tumor beta—normal beta) on CTCF in-boundary motifs on a cohort of Breast Cancer patients. As previously reported, methylation peaks are found on the CpG dinucleotides. Evident in the figure, active in-boundary motifs are frequently over-methylated in tumour samples compared to normal samples.

In several cancer types we observed hypermethylation on in-boundary motifs. Specifically, the phenomenon is significant in Breast Cancer (BRCA), Head and Neck Squamous Cell Carcinoma (HNSC), Kidney Renal Papillary Cell Carcinoma (KIRP), Uterine Corpus Endometrial Carcinoma (UCEC), Lung Squamous Cell Carcinoma (LUSC) and Skin Cutaneous Melanoma (SKCM). In order to validate that such phenomenon is specific of in-boundary junctions and not due to an overall genome over-methylation of these tumors, we designed a bootstrap test. Specifically, we calculated Δ*β* = (*β*_*tumor*_ − *β*_*normal*_) for all the probes. We then sampled an equal number of probes from in-boundary and off-boundary motifs and calculated the mean of these probes. We repeated the sampling 10,000 times and counted how many times the mean Δ*β* within in-boundary is greater than the one within off-boundary motifs. The empirical p-values are 0 in Breast Cancer (BRCA), Head and Neck Squamous Cell Carcinoma (HNSC), Uterine Corpus Endometrial Carcinoma (UCEC), Lung Squamous Cell Carcinoma (LUSC), Skin Cutaneous Melanoma (SKCM), Bladder Urothelial Cancer (BLCA), Liver Hepatocellular Carcinoma (LIHC) and Thyroid Cancer (THCA), indicating that in these cancer types the Δ*β* is significantly larger within in-boundary than off-boundary motifs, i.e., these motifs are more methylated than off-boundary ones. Conversely, in the cases of Kidney Renal Papillary Cell Carcinoma (KIRP), Kidney Renal Clear Cell Carcinoma (KIRC), Lung Adenocarcinoma (LUAD) and Prostate Adenocarcinoma (PRAD), we obtain a p-value equal to 1, implying that in these cancers, the in-boundary motifs are significantly less methylated than the off-boundary motifs.

To evaluate whether over-methylated motifs are conserved across different cancer types, we considered the five most over-methylated tumour types according to the boundaries defined for the hESC cell line (see Table 4, Left subplot): Breast Cancer, Uterine Corpus Endometrial Carcinoma, Head and Neck Squamous Cell Carcinoma, Kidney Renal Papillary Cell Carcinoma and Lung Squamous Cell Carcinoma. For each cancer type, we identified the set of active in-boundary motifs with the strongest methylation difference between tumour and normal, i.e. abs(tumour beta—normal beta)> 0.2, and we computed the overlap across all 5 cancer types. Although the sets of differentially methylated active in-boundary motifs are different for each tumour, they have strongly significant overlaps, as shown in Fig L in S1 File. Significance is computed by mean of a hypergeometric test, as previously discussed. Results are reported in Table H in S1 File.

**Table 4:** **a) Empirical p-values indicating the significance of the over-methylation of active, in-boundary CTCF motifs** compared to off-boundary random positions in the genome, computed by means of a permutation test. **b) p-values indicating the enrichment of CNA mutations on active in-boundary motifs with respect to off-boundary motis** in tumour samples. P-values have been computed using the chi-square test on a *P* × 2 contingency table, where *P* is the number of patients. In brackets we report the median size of the CNA-mutated regions for the given cancer.

Tumour	hESC	MCF7	Hnisz
**(a)Methylation**			
**BRCA**	<0.0001	<0.0001	<0.0001
**HNSC**	<0.0001	<0.0001	<0.0001
**KIRP**	1.0	1.0	1.0
**UCEC**	<0.0001	<0.0001	<0.0001
**LUSC**	<0.0001	<0.0001	<0.0001
**SKCM**	<0.0001	<0.0001	<0.0001
**PRAD**	1.0	1.0	1.0
**BLCA**	<0.0001	<0.0001	<0.0001
**LIHC**	<0.0001	<0.0001	<0.0001
**THCA**	<0.0001	0.9737	0.2207
**LUAD**	0.2523	1.0	1.0
**KIRC**	0.6821	1.0	1.0
**(b)CNA**			
**LUAD** (66k)	<0.0001	<0.0001	<0.0001
**OV** (69k)	<0.0001	<0.0001	<0.0001
**BRCA** (42k)	0.0126	<0.0001	<0.0001
**LIHC** (36k)	0.1485	0.0048	0.0992
**UCEC** (26k)	0.1618	0.0276	0.0227
**BLCA** (47k)	0.3419	<0.0001	0.0056
**PRAD** (15k)	0.4061	0.0257	0.0942
**GBM** (16k)	0.8046	0.0001	0.3685
**HNSC** (37k)	0.8140	<0.0001	0.9749
**LUSC** (38k)	0.9328	<0.0001	0.7454
**KIRP** (14k)	1.0000	0.4314	0.3756

### CNA analysis

Copy number alterations (CNA) are a main tumorigenic driver in many cancer types [47]. In the context of neighbourhood dysregulation, recent work has reported tandem duplications intersecting with a TAD that led to de novo 3D contact domain formation. The domain rearrangement affected a lineage-specific super-enhancer, resulting in high-level gene activation [48]. The same work revealed that TAD boundary intersecting deletions are associated with IRS4 dysregulation (a gene often over-expressed in different cancer types) in sarcoma and squamous cancers. Motivated by these findings, we investigate here whether copy number alterations may contribute to cancer phenotypes by disrupting topologically associated domain boundaries.

To evaluate the effect of CNAs on a neighbourhood’s dysregulation, we analysed CNA data for 11 different types of cancer provided by ICGC. We considered a genomic region mutated, i.e. either deleted or amplified, if it has an (absolute) mean value greater than 0.2 [49]. A major difference with respect to somatic alterations and methylation changes is that CNAs are not local alterations, but can span large areas of a chromosome. A large CNA that affects a sizeable portion of a chromosome is likely to induce gene dysregulation by a combination of mechanisms, including oncogene and/or tumor suppression dysregulation, as well as insulated neighbourhood dysregulation. Such multi-factorial dysregulatory effect is less likely to be present in small CNAs. To quantify this effect, we stratified CNAs by size splitting them in 4 groups according to the 25%, 50% (median), 75% and 100% percentiles of the size distribution of each cancer type. Note that the 100% percentile includes all CNAs available for analysis for each cancer.

#### Copy number alterations of active in-boundary vs off-boundary motifs

To test the potential association between cancer and copy-number mutation affecting insulated neighbourhoods, we compared the distribution of CNA-mutated regions overlapping in-boundary motifs with the distribution of regions overlapping off-boundary motifs *in tumour samples*. In practice, for each cancer, we built a *P* × 2 contingency table where each row represents a patient. We reported in Table 4b the p-values obtained by performing a chi-square test on such contingency tables, using the median of the CNA size distribution as a cutoff for CNA size. Results for all 4 cutoffs can be found in Tables I and J in S1 File. Interestingly, for all three the neighbourhood datasets and for all size cutoffs (with the exception of the 25th percentile), we obtained significant results for breast cancer, lung adenocarcinoma, and ovarian cancer. The non-significance of the results for small cutoffs may be explained by the number of patients considered (reported in brackets in Tables I and J in S1 File). Indeed, for the smaller cutoffs, we retained a small number of patients suggesting that the high p-values may be due to low statistical power.

In recent work [50], principal component analysis (PCA) on CNA data was performed on various cancer types, and Ovarian, Lung and Breast (basal subtype) cancers were found to have a similar *signature* characterised by a higher degree of copy number alterations compared to other types of cancer. Our results are in agreement with these findings: we observe that ovarian, lung and breast cancers have an enrichment of CNAs overlapping active, in-boundary motifs, which might potentially lead to the dysregulation of key cancer drivers. Note that we obtained our results by focusing on CNAs with an absolute segment mean greater than 0.2 and by considering only active motifs, while [50] used *gene* mean CNA values, which do not in general co-locate with motifs. Remarkably, we find similar patterns as in [50] while looking at different positions in the genome (active, in-boundary motifs versus coding genes). This strongly suggests that the dysregulation of neighbourhood boundaries through the accumulation of CNAs is not a fortuitous occurrence, but could instead be a driver mechanism in these cancer types.

## Results and discussion

Cancer arises as a result of heterogeneous molecular mechanisms that lead to the silencing of tumour suppressor genes and activation of oncogenes. It has been reported in the past that disruption of constitutive insulated neighbourhoods leads to oncogene activation [22, 23, 31].

Recent reviews and opinion papers have highlighted the central role of boundary disruption leading to genomic rearrangement in cancer development [51–55], and several recent articles have connected the disruption of constitutive neighbourhoods to oncogene dysregulation in specific tumours.

In [22] for instance, the loss of one boundary enables a constitutive enhancer to interact aberrantly with PDGFRA, a prominent oncogene in glioma. In [23], the loss of one boundary is linked to the activation of TAL1, a proto-oncogene in acute lymphoblastic leukemia. In [31], mutations of one insulator region identified as a melanoma driver are associated with the upregulation of TGFB1, although another study on melanoma could not find evidence of gene expression enrichment [28]. Other studies have described gene expression changes in the proximity of mutation hotspots at CTCF binding sites in gastrointestinal cancer [30]. While each of these results individually suggests an important role for the dysregulation of some constitutive neighbourhoods in specific tumors, a conclusive pan-cancer analysis is not yet available.

In this work, we present a systematic pan-cancer analysis of the alterations of neighbourhood boundaries that actively support DNA looping and the constitution of insulated neighbourhoods. We concentrate on CTCF motif occurrences that are confirmed by ChIP-seq experiments in three cell lines, and we further divide them as in-boundary vs off-boundary by using boundary regions as identified by biologically coherent ChIA-PET experiments. Specifically, we have investigated the alteration of CTCF bindings by means of three different genomic alterations: the acquisition of somatic mutations, abnormal methylation and copy number alterations. All three mechanisms can disrupt or completely eliminate a motif, hence preventing the binding of CTCF and compromising the integrity of its associated neighbourhood. In the case of somatic mutations, we opted for a descriptive unbiased approach where we summarised all mutations falling within a motif. Our result complements existing studies that have highlighted the prevalence of mutation enrichments at CTCF sites and provides a novel insights about the impact of methylation and copy number alteration on boundary dysregulation, which have not yet been systematically analysed.

Table 5 summarises our analysis: we find that somatic mutations, methylation, and copy number variations are significantly enriched in the neighbourhood boundaries in some specific cancer types. Specifically, we observed an enrichment of somatic mutations in at least two insulated neighbourhood datasets in eight cancer types. A systematic analysis of the type of mutation observed in each cancer type (Fig 5) reveals the appearance of mutation peaks on specific positions of the motif, which are not present on off-boundary CTCF motifs. We also observe a very significant overlap of frequently mutated active boundaries on these five cancers (Table G in S1 File), confirming that mutations in boundary do not happen at random. A positive selection of transversions versus transitions seems to be prevalent in most cancer types. We speculate that transversions might be positively selected, as they have a slightly larger effect on DNA shape and transcription factor binding than transitions [41].

**Table 5:** Significant alterations in cancer types. For each of the three analyses we performed (enrichment of somatic mutations, DNA methylation and CNA) we report the result corresponding to each of the three datasets of insulated neighbourhoods: hESC (**h**), MCF7 (**M**) and Hnisz (**H**). A *dash* indicates that data is not available for the specific cancer type, Y that the alteration is significant (p-value <.05), N that the alteration is not significant (p-value ≥ .05). For methylation Y(+) indicates that a significant hyper-methylation is observed, Y(-) that a significant hypo-methylation is observed.

	Somatic mut.			DNA meth.			CNA		
Tumour	h	M	H	h	M	H	h	M	H
**BLCA**	-	-	-	Y(+)	Y(+)	Y(+)	N	Y	Y
**BOCA**	-	-	-	-	-	-	-	-	-
**BRCA**	N	Y	Y	Y(+)	Y(+)	Y(+)	Y	Y	Y
**BTCA**	-	-	-	-	-	-	-	-	-
**COCA**	N	Y	Y	-	-	-	-	-	-
**EOPC**	-	-	-	-	-	-	-	-	-
**ESAD**	Y	Y	Y	-	-	-	-	-	-
**GACA**	N	Y	Y	-	-	-	-	-	-
**GBM**	-	-	-	-	-	-	N	Y	N
**HNSC**	-	-	-	Y(+)	Y(+)	Y(+)	N	Y	N
**KIRC**	-	-	-	N	Y(-)	Y(-)	-	-	-
**KIRP**	-	-	-	Y(-)	Y(-)	Y(-)	N	N	N
**LIHC**	-	-	-	Y(+)	Y(+)	Y(+)	N	Y	N
**LIRI**	Y	Y	Y	-	-	-	-	-	-
**LUAD**	-	-	-	N	Y(-)	Y(-)	Y	Y	Y
**LUSC**	-	-	-	Y(+)	Y(+)	Y(+)	N	Y	N
**MALY**	Y	Y	Y	-	-	-	-	-	-
**MELA**	Y	Y	Y	-	-	-	-	-	-
**OV**	-	-	-	-	-	-	Y	Y	Y
**PACA**	-	-	-	-	-	-	-	-	-
**PRAD**	-	-	-	Y(-)	Y(-)	Y(-)	N	Y	N
**RECA**	-	-	-	-	-	-	-	-	-
**SKCA**	Y	Y	Y	-	-	-	-	-	-
**SKCM**	-	-	-	Y(+)	Y(+)	Y(+)	-	-	-
**THCA**	-	-	-	Y(+)	N	N	-	-	-
**UCEC**	-	-	-	Y(+)	Y(+)	Y(+)	N	Y	Y

Regarding methylation, we tested in-boundary CTCF motifs in twelve cancer types and we found that seven of them are significantly hyper-methylated while four of them are significantly hypo-methylated. We also observe a very significant overlap of frequently over-methylated active boundaries on these cancers, confirming that also the over-methylated dysregulation of boundaries does not happen by random chance. Finally, we observe that copy number alterations significantly overlap with active junctions in four cancer types, namely in Breast Cancer (BRCA), Lung Adenocarcinoma (LUAD), Lung Squamous Carcinoma (LUSC) and Ovarian Cancer (OV), all of them cancers where important oncogenic CNA signatures have been identified [50, 56].

In S2 File we report pan-cancer integrated information; rows describe CTCF motif occurrences indexed by genomic coordinates, their active-inactive state, their overlap with neighborhood boundaries and for each tumor their counts of mutations and hyper methylated probes.

Unfortunately, we do not have access to all studied data types for all considered cancers, and Breast Cancer is the only tumor type for which we have data about all three mutagenic processes. Remarkably, breast cancer appears to be significantly enriched in all 3 considered boundary dysregulation mechanims. We note that the amplification of estrogen response elements has been reported to be generated by abnormal long-range chromatin interactions [57], further highlighting the importance of maintaining a correctly regulated genome architecture.

To summarise, although additional analysis and experimental validation are needed to understand the causal relationship between these observations and the abnormal modifications of the genome 3D structure, our research presents a systematic observation of the relationships between topological boundaries and genome alterations. One of the immediate outcomes of our work is a method for the identification of deregulated junctions, enabling a detailed, tumour-specific analysis on a smaller scale, as in [22, 23, 27, 28, 30].

## Supporting information

S1 File Supplementary material, containing Tables A-J and Figs A-L. (PDF)

S2 File Pancancer integrated information; rows describe CTCF motif occurrences indexed by genomic coordinates, their activeinactive state, their overlap with neighborhood boundaries and for each tumor their counts of mutations and hyper methylated probes. (XLS)

S3 File Readme file for S2 File. (RTF)

S1 Notebook Jupyter Notebook for mutation analysis. (IPYNB)
