# Supplementary material for: Pan-cancer analysis of somatic mutations and epigenetic alterations in insulated neighbourhood boundaries
Source: PLoS One. 2020 Jan 16;15(1):e0227180. doi: 10.1371/journal.pone.0227180 (PMC6964824; doi:10.1371/journal.pone.0227180)
Supplement: S1 File — (PDF) [file pone.0227180.s001.pdf]

Supplementary Information

Pan-cancer analysis of somatic mutations and epigenetic alterations in insulated neighbourhood boundaries

Pietro Pinoli, Eirini Stamoulakatou, An-Phi Nguyen, María Rodríguez Martínez, Stefano Ceri

Code	Tumor
**BLCA**	Bladder Urothelial Carcinoma
**BOCA**	Bone Cancer
**BRCA**	Breast Cancer
**BTCA**	Biliary Tract Cancer
**COCA**	Colorectal Cancer
**EOPC**	Early Onset Prostate Cancer
**ESAD**	Esophageal Adenocarcinoma
**GACA**	Gastric Cancer
**GBM**	Glioblastoma Multiforme
**HNSC**	Head and Neck Squamous Cell Carcinoma
**KIRC**	Kidney Renal Clear Cell Carcinoma
**KIRP**	Kidney Renal Papillary Cell Carcinoma
**LIHC**	Liver Hepatocellular Carcinoma
**LIRI**	Liver Cancer
**LUAD**	Lung Adenocarcinoma
**LUSC**	Lung Squamous Carcinoma
**MALY**	Malignant Lymphoma
**MELA**	Skin Cancer
**OV**	Ovarian Cancer
**PACA**	Pancreatic Cancer Endocrine Neoplasms
**PRAD**	Prostate Adenocarcinoma
**RECA**	Renal Cell Cancer
**SKCA**	Skin Adenocarcinoma
**SKCM**	Skin Cutaneous Melanoma
**THCA**	Thyroid Carcinoma
**UCEC**	Uterine Corpus Endometrial Carcinoma

Table A: List of considered cancer types.

**Code**	**Samples**	**mutations**
**MELA**	183	25189055
**ESAD**	301	10593950
**SKCA**	100	7969172
**LIRI**	258	3994848
**BRCA**	301	3973993
**MALY**	241	3274938
**PACA**	252	1475766
**OV**	93	1277942
**BTCA**	71	1188089
**RECA**	95	761813
**GACA**	37	564986
**EOPC**	202	531029
**COCA**	26	281447
**BOCA**	64	210646

Table B: Number of patients and mutations ICGC studies used for mutation analysis.

**Tumor**	**hESC**
**BRCA**	1.0
**MELA**	1.0
**LIRI**	1.0
**COCA**	1.0
**ESAD**	1.0
**GACA**	0.9989
**SKCA**	0.9671
**BOCA**	0.9658
**OV**	0.7913
**MALY**	0.7995
**RECA**	0.7591
**EOPC**	0.5671
**BTCA**	0.2216

Table C: Empirical p-values obtained through a permutation test comparing the enrichment of mutations of active CTCF motifs within and outside promoters for all cancer types. No p-value is found to be significant, corroborating the finding that active boundaries tend to be enriched in mutations in cancer genomes due to cancer-specific mechanisms, and not because of their potential overlap with promoter regions.

Code	Samples	Total probes
**KIRC**	160	49541968
**BRCA**	87	26910127
**THCA**	56	17335354
**PRAD**	50	15479766
**HNSC**	50	15468019
**LIHC**	50	15458811
**KIRP**	45	13923331
**LUSC**	40	12381681
**UCEC**	33	10111501
**LUAD**	23	7113353
**BLCA**	20	6193588

Table D: Number of patients for which at least 20 matched tumour and normal methylation samples are available in TCGA. The number of probes for each cancer type is also reported.

	**Total**	**Overlap exons**	**Overlap promoters**	**Overlap enhancers**
**Methylation array probes**	309851	100097 (33%)	167956 (54%)	34230 (11%)
**Active in-boundary motifs overlapping probes in hESC**	342	85 (25%)	143 (42%)	13 (4%)
**Active in-boundary motifs overlapping probes in MCF7**	274	56 (20%)	122 (44%)	7 (3%)
**Active in-boundary motifs overlapping probes in Hnisz**	314	77 (25%)	136 (43%)	10 (3%)

Table E: Summary of the datasets used for methylation analysis.

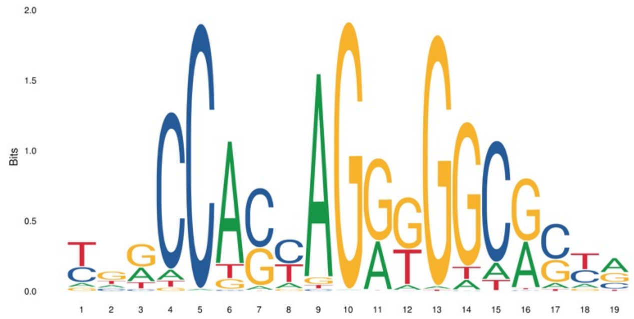

Figure A: Jaspar MA0139.1 CTCF motif (19 base pairs).

Code	Samples	#segments (normal)	Avg. segments mean (normal)	#segments (tumor)	Avg. segments mean (tumor)
**BLCA**	236	88366	-0.43	68665	-0.75
**BRCA**	1000	193326	-0.68	273110	-0.63
**GBM**	482	99716	-0.90	144650	-0.91
**HNSC**	452	98707	-0.54	95786	-0.65
**KIRP**	209	45922	-0.67	47504	-0.70
**LIHC**	291	56888	-0.70	63504	-0.64
**LUAD**	429	96323	-0.66	188009	-0.31
**LUSC**	400	107445	-0.65	129383	-0.59
**OV**	538	145873	-0.59	255759	-0.54
**PRAD**	361	119610	-0.49	82581	-0.68
**THCA**	432	76442	-0.70	75668	-0.67
**UCEC**	487	135623	-0.43	133589	-0.59

Table F: Summary of the datasets used for CNA analysis.

Tumour A	Tumour B	Intersection	pvalue
**ESAD**	**LIRI**	297	2.1e-87
**ESAD**	**MELA**	539	5.6e-27
**ESAD**	**SKCA**	208	1.5e-15
**ESAD**	**BRCA**	176	4.5e-30
**LIRI**	**MELA**	294	2.0e-24
**LIRI**	**SKCA**	114	7.5e-13
**LIRI**	**BRCA**	108	1.3e-26
**MELA**	**SKCA**	491	5.1e-81
**MELA**	**BRCA**	204	4.2e-10
**SKCA**	**BRCA**	92	4.9e-11

Table G: **Pairwise significance of the overlap between mutated in-boundary motifs across several tumour types** using hESC insulated neighborhoods. The column *Intersection* reports the number of motifs that are simultaneously mutated in both pairs of tumours. The *p-value* is computed by a hypergeometric test as the probability of observing a value greater or equal to *Intersection*.

Tumour A	Tumour B	Intersection	p-value
**BRCA**	**HNSC**	132	2.6e-257
**BRCA**	**KIRP**	126	9.1e-235
**BRCA**	**LUSC**	120	1.7e-227
**BRCA**	**UCEC**	144	3.4e-258
**HNSC**	**KIRP**	110	1.1e-209
**HNSC**	**LUSC**	111	8.5e-222
**HNSC**	**UCEC**	128	6.7e-244
**KIRP**	**LUSC**	102	1.1e-191
**KIRP**	**UCEC**	116	1.0e-204
**LUSC**	**KIRP**	119	1.32-229

Table H: **Pairwise significance of the overlap between differentially methylated in-boundary motifs across several tumour types** using hESC insulated neighborhoods. The column *Intersection* reports the number of motifs that are simultaneously differentially methylated in both pairs of tumours. The *p-value* is computed by a hypergeometric test as the probability of observing a value equal or greater than *Intersection*.

Cancer Type	Size cutoff	HESC	MCF7	HNISZ
LUAD	9688	0.6599 (11)	0.3149 (19)	0.0060 (20)
	66164	0.0000 (121)	0.0000 (250)	0.0000 (164)
	467907	0.0000 (410)	0.0000 (435)	0.0000 (424)
	218512014	0.0000 (440)	0.0000 (444)	0.0000 (443)
BOCA	7399	0.4043 (2)	0.3278 (2)	0.7566 (2)
	14899	0.1362 (3)	0.0249 (3)	0.5561 (3)
	33099	0.0008 (4)	0.0123 (4)	0.0047 (4)
	243173599	0.0000 (68)	0.0000 (69)	0.0000 (68)
KIRP	13546	1.0000 (1)	0.4314 (2)	0.3756 (3)
	168288	0.0997 (75)	0.0000 (155)	0.0000 (112)
	162935357	0.0000 (210)	0.0000 (210)	0.0000 (210)
GBM	15540	0.8046 (10)	0.0001 (34)	0.3685 (37)
	218960	0.0000 (474)	0.0000 (528)	0.0000 (499)
	135433945	0.0000 (555)	0.0000 (557)	0.0000 (556)
LUSC	37848	0.9328 (31)	0.0000 (102)	0.7454 (58)
	917109	0.0000 (415)	0.0000 (417)	0.0000 (416)
	135433945	0.0000 (420)	0.0000 (420)	0.0000 (419)
SKCA	4464	0.6067 (4)	0.3471 (4)	0.1072 (4)
	111821	0.0026 (21)	0.0542 (41)	0.0000 (32)
	249229885	0.0000 (100)	0.0000 (100)	0.0000 (100)
BLCA	5543	1.0000 (1)	0.6968 (2)	1.0000 (1)
	47202	0.3419 (84)	0.0000	0.0056 (50)
	986442	0.0000 (1009)	0.0000	0.0000 (263)
	249230398	0.0000 (1028)	0.0000	0.0000 (276)

Table I: **P-values of the chi-square test for enrichment of CNA-mutation of in-boundary vs off-boundary motifs.** (Part 1) Results are shown for each cancer types and for 4 possible size cutoffs, i.e. 25th, (median) 50th, 75th, and 100th percentiles of the CNA size distribution. If for a cancer type there are less than 4 rows, it means that we did not run the test for the smaller cutoffs because of lack of samples to run the chi-square test. In brackets we report the number of patients retained for the test after cutoff. Red cells highlight tests with less than 10 patients.

Cancer Type	Size cutoff	HESC	MCF7	HNISZ
BRCA	4361	1.0000 (1)	0.1129 (4)	0.2826 (3)
	42236	0.0126 (84)	0.0000 (322)	0.0122 (209)
	808789	0.0000 (1009)	0.0000 (1027)	0.0000 (1014)
	249230397	0.0000 (1028)	0.0000 (1033)	0.0000 (1030)
UCEC	25869	0.1618 (12)	0.0276 (70)	0.0227 (36)
	406255	0.0000 (438)	0.0000 (484)	0.0000 (465)
	243179358	0.0000 (490)	0.0000 (501)	0.0000 (503)
THCA	53431	0.0037 (27)	0.0000 (135)	0.8100 (53)
	249230398	0.0000 (362)	0.0000 (420)	0.0000 (407)
HNSC	36853	0.8140 (13)	0.0000 (90)	0.9749 (39)
	906018	0.0000 (446)	0.0000 (458)	0.0000 (454)
	208033911	0.0000 (458)	0.0000 (461)	0.0000 (460)
OV	5968	0.5108 (6)	0.1634 (9)	0.4896 (9)
	68678	0.0000 (340)	0.0000 (461)	0.0000 (401)
	1355458	0.0000 (565)	0.0000 (565)	0.0000 (565)
	148719764	0.0000 (565)	0.0000 (565)	0.0000 (565)
PRAD	14812	0.4061 (3)	0.0257 (6)	0.0942 (6)
	147844	0.0048 (185)	0.0000 (288)	0.0000 (236)
	243179358	0.0000 (362)	0.0000 (374)	0.0000 (370)
LIHC	36228	0.1485 (19)	0.0048 (56)	0.0992 (36)
	730141	0.0000 (289)	0.0000 (299)	0.0000 (297)
	165511172	0.0000 (302)	0.0000 (304)	0.0000 (304)

Table J: **P-values of the chi-square test for enrichment of CNA-mutation of in-boundary vs off-boundary motifs.** (Part 2) Results are shown for each cancer types and for 4 possible size cutoffs, i.e. 25th, (median) 50th, 75th, and 100th percentiles of the CNA size distribution. If for a cancer type there are less than 4 rows, it means that we did not run the test for the smaller cutoffs because of lack of samples to run the chi-square test. In brackets we report the number of patients retained for the test after cutoff. Red cells highlight tests with less than 10 patients.

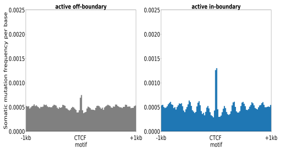

(a) MELA

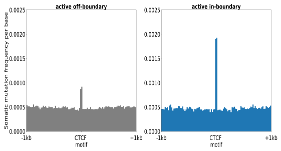

(b) ESAD

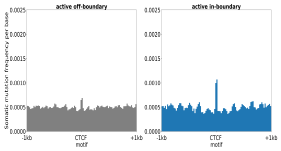

(c) SKCA

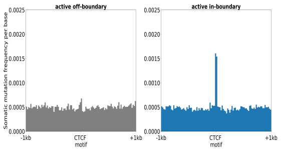

(d) LIRI

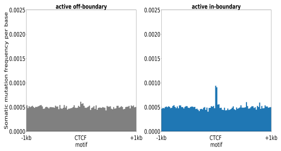

(e) BRCA

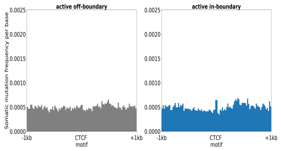

(f) MALY

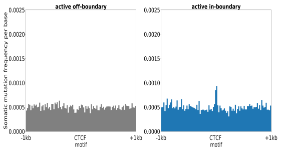

(g) PACA

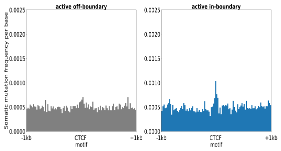

(h) OV

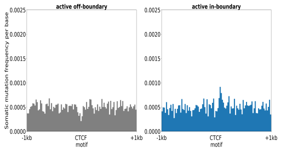

(i) BTCA

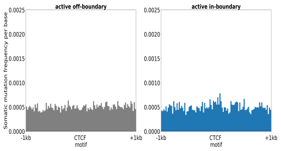

(j) RECA

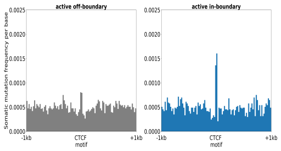

(k) GACA

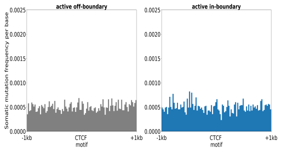

(l) EOPC

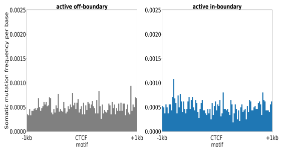

(m) COCA

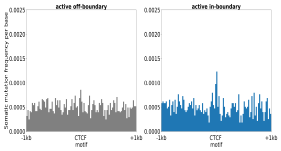

(n) BOCA

Figure B: Enrichment of mutations on junctions across different cancer types; boundaries are taken from the ChIA-PET experiment on the MCF7 cell line.

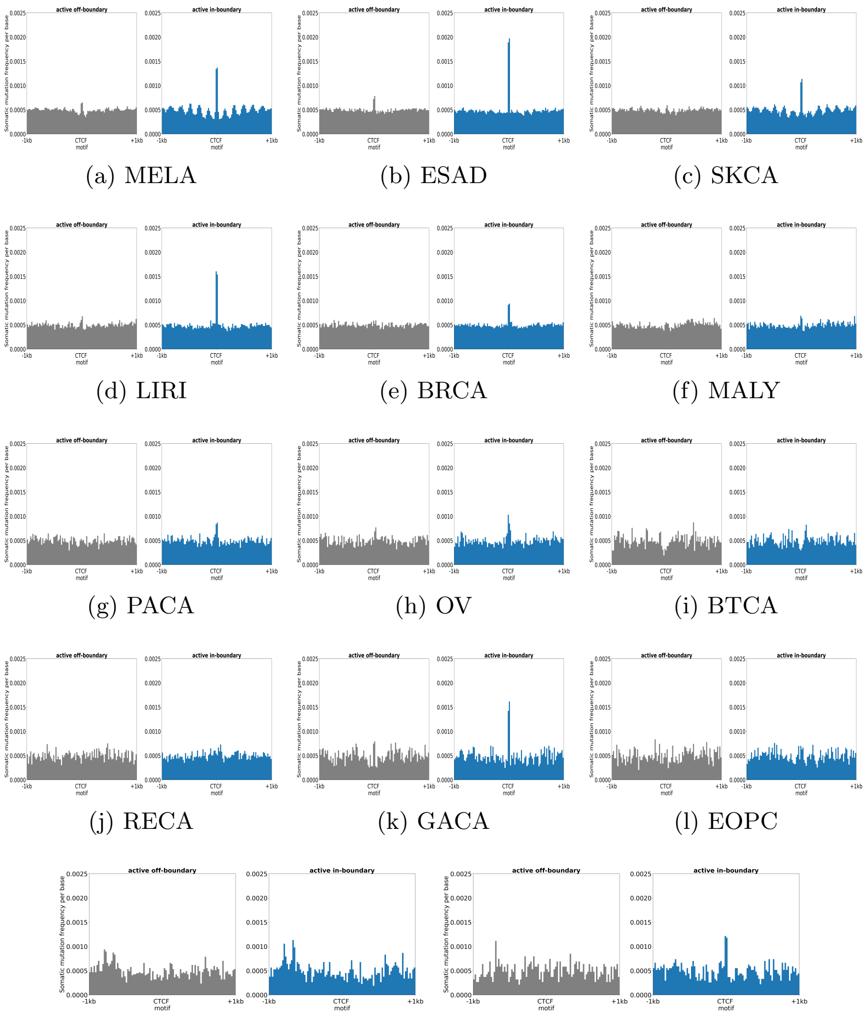

(a) MELA (b) ESAD (c) SKCA

(d) LIRI (e) BRCA (f) MALY

(g) PACA (h) OV (i) BTCA

(j) RECA (k) GACA (l) EOPC

(m) COCA (n) BOCA

Figure C: Enrichment of mutations on junctions across different cancer types; boundaries are taken from the ChIA-PET experiment on the hESC cell line.

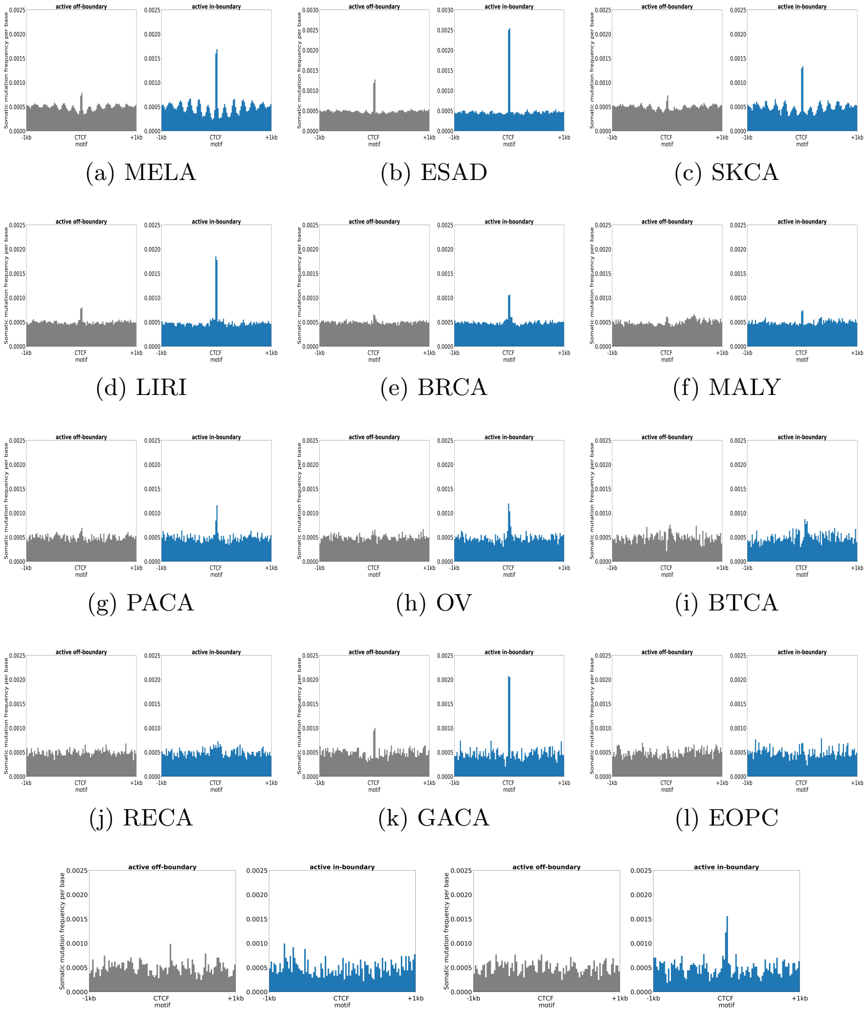

(a) MELA (b) ESAD (c) SKCA

(d) LIRI (e) BRCA (f) MALY

(g) PACA (h) OV (i) BTCA

(j) RECA (k) GACA (l) EOPC

(m) COCA (n) BOCA

Figure D: Enrichment of mutations on junctions across different cancer types; boundaries are taken from the set of insulated neighborhoods published by Hnisz et al.

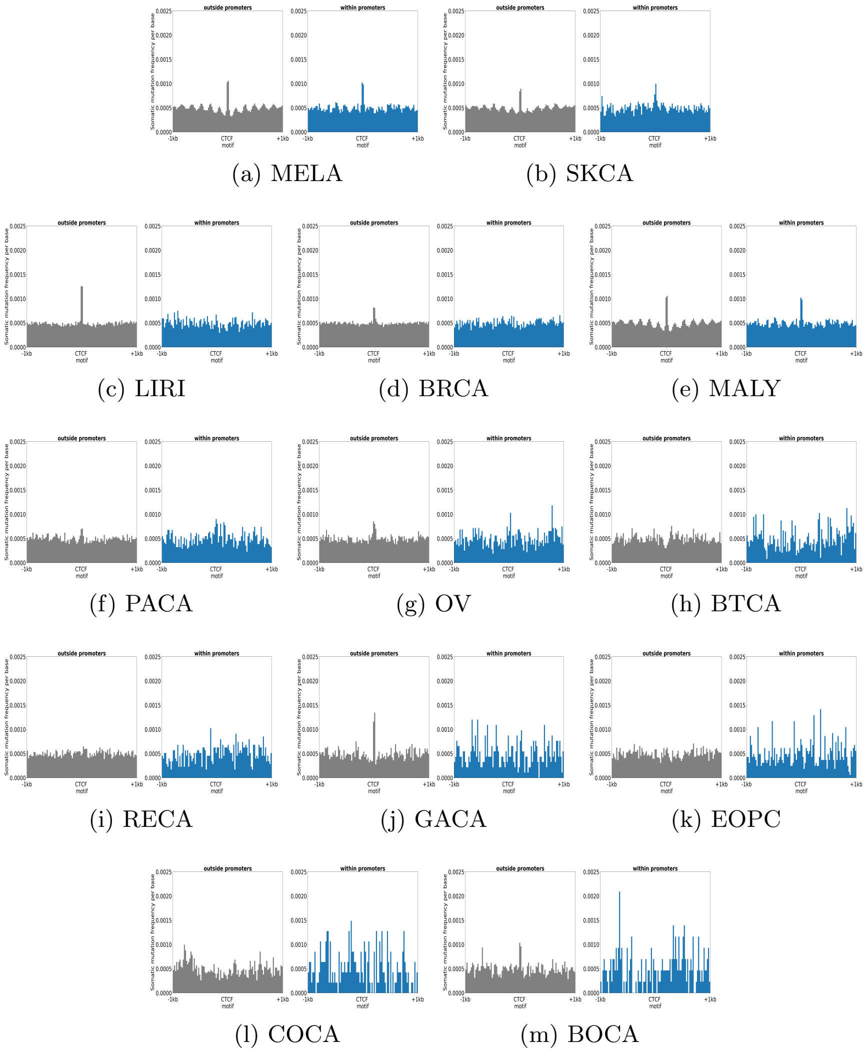

Figure E: Enrichment of mutations on promoters across different cancer types; boundaries are taken from a ChIA-PET experiment on a hESC cell line.

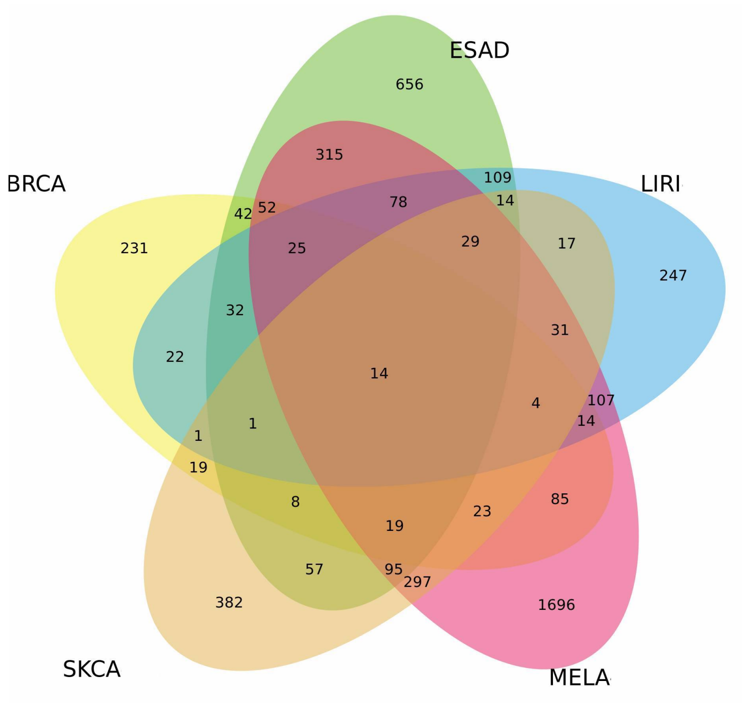

Figure F: **Overlap of mutated in-boundary motifs across several tumour types** using hESC neighbourhoods. Most of the in-boundary motifs are mutated in at least two different cancer types, with 14 of them presenting mutations in all of the 5 considered cancer types.

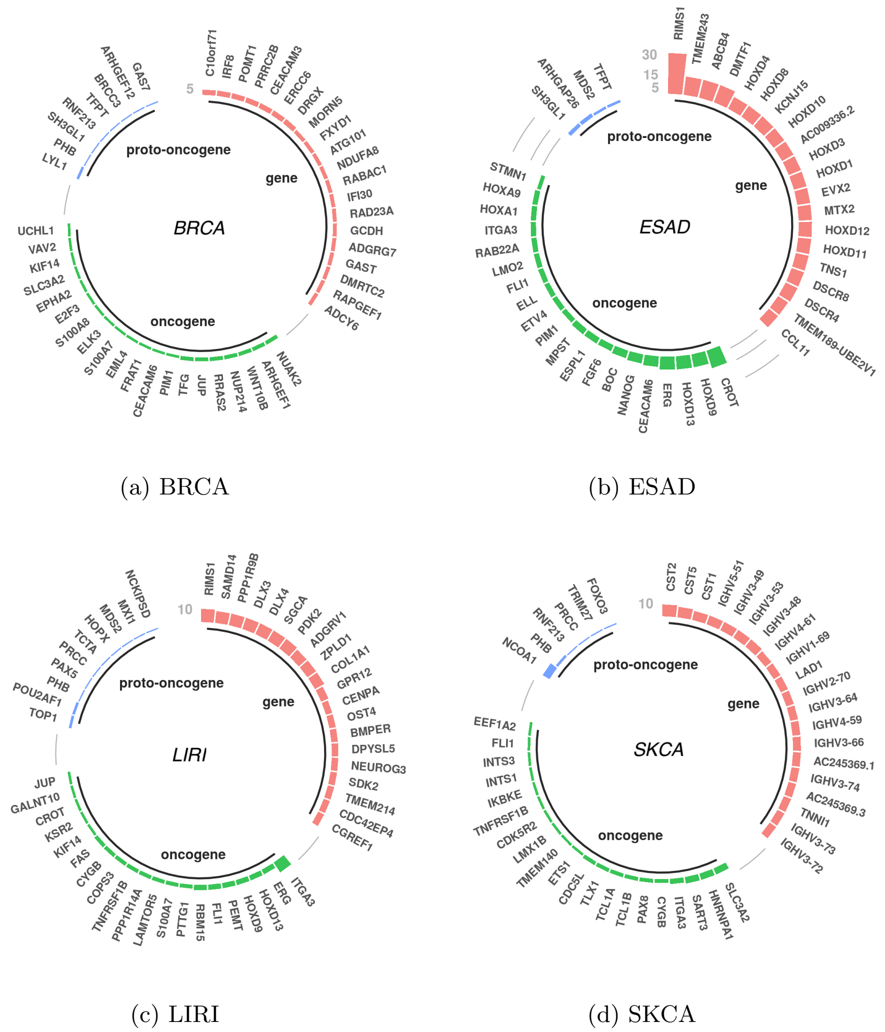

(a) BRCA

(b) ESAD

(c) LIRI

(d) SKCA

Figure G: Genes close to mutated active in-boundary CTCF motifs.

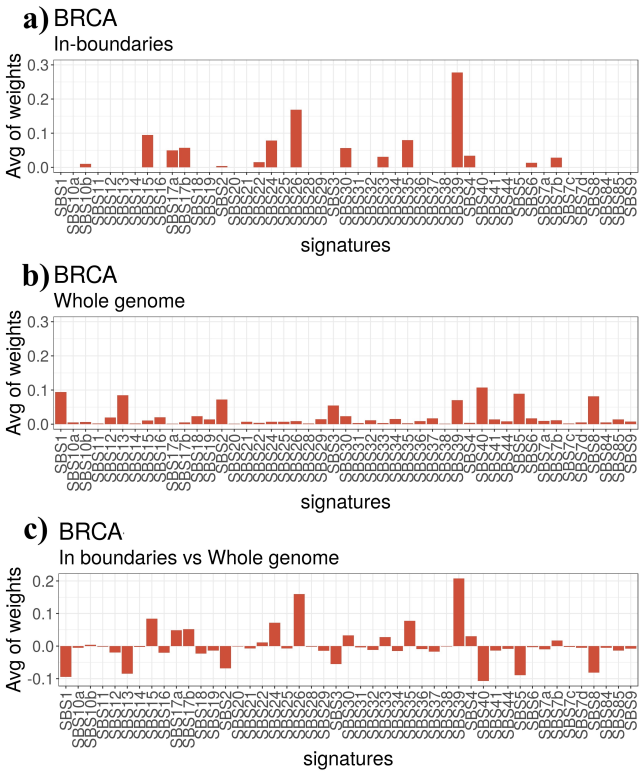

Figure H: The average contributions or weights (i.e., exposures) of mutational signatures for the BRCA dataset. Signature refitting was done based on mutations falling a) in-boundaries motifs, b)in the whole genome, and c) the difference between the two.

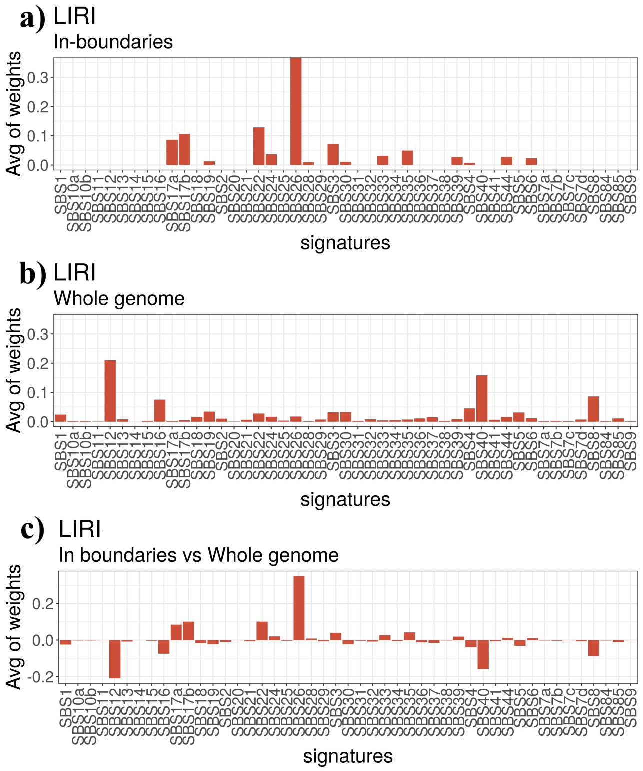

Figure I: The average contributions or weights (i.e., exposures) of mutational signatures for the LIRI dataset. Signature refitting was done based on mutations falling a) in-boundaries motifs, b)in the whole genome, and c) the difference between the two.

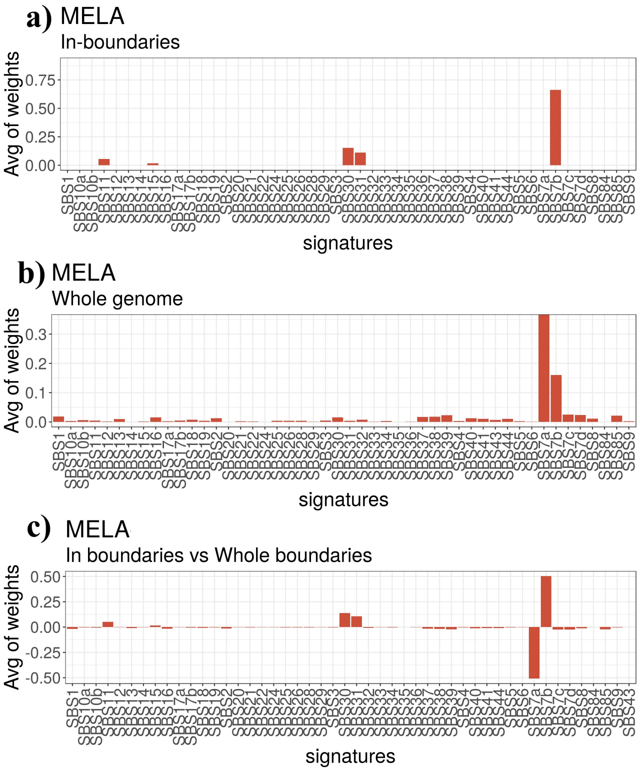

Figure J: The average contributions or weights (i.e., exposures) of mutational signatures for the MELA dataset. Signature refitting was done based on mutations falling a) in-boundaries motifs, b)in the whole genome, and c) the difference between the two.

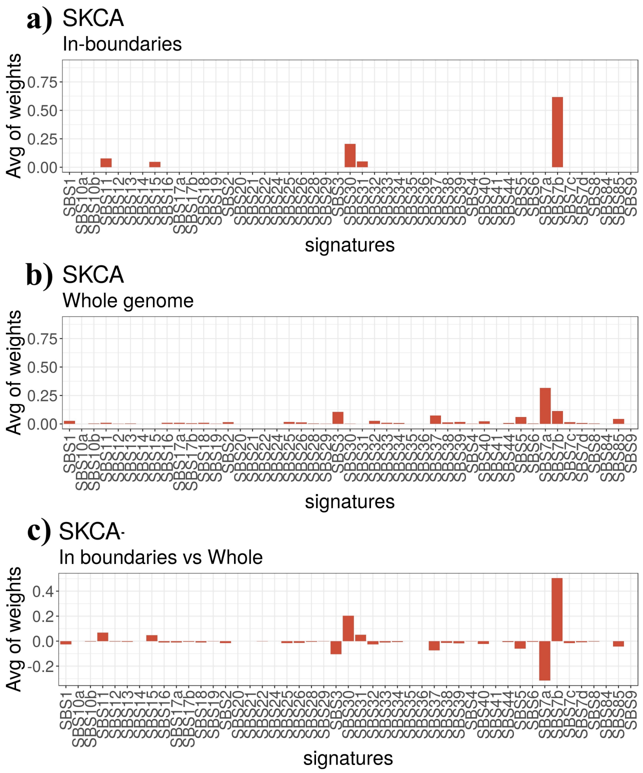

Figure K: The average contributions or weights (i.e., exposures) of mutational signatures for the SKCA dataset. Signature refitting was done based on mutations falling a) in-boundaries motifs, b)in the whole genome, and c) the difference between the two.

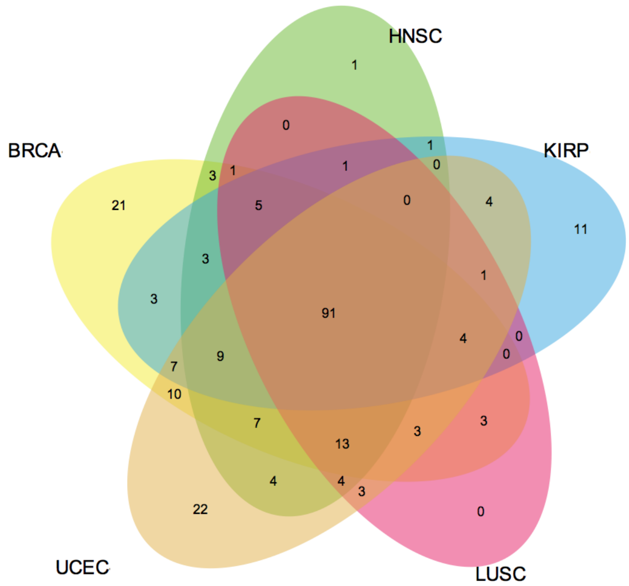

Figure L: Dysregulated methylated active in-boundary motifs using the hESC neighbourhoods that are shared across five tumours.
