# Supplementary material for: Pan-cancer analysis of somatic mutations and epigenetic alterations in insulated neighbourhood boundaries
Source: PLoS One. 2020 Jan 16;15(1):e0227180. doi: 10.1371/journal.pone.0227180 (PMC6964824; doi:10.1371/journal.pone.0227180)
Supplement: S3 File — (RTF) [file pone.0227180.s003.rtf]

SUPPLEMENTARY MATERIAL – Table of Mutations and Methylations for several Cancer TypesPan-cancer analysis of somatic mutations and epigenetic alterations in insulated neighbourhood boundariesPietro Pinoli, Eirini Stamoulakatou, An-Phi Nguyen, Maria Rodriguez Martinez, and Stefano CeriMatrix descriptionChr: the chromosome of the CTCF motifStart: the starting base of the 19 base CTCF motifStop: the end base of the 19 base CTCF motifactive_Hnisz: if is 1 then the motif is active in the Hnisz cell lines, else 0 (inactive)active_hESC: if is 1, then the motif is active in the hESC cell line, else 0 (inactive)active_MCF7: if is 1, then the motif is active in the MCF7 cell line, else 0 (inactive)in_Hnisz: 1 if the motif is inside a boundary in the Hnisz cell lines, else 0 (out)in_hESC: 1 if the motif is inside a boundary in the hESC cell line, else 0 (out)in_MCF7: 1 if the motif is in a boundary in the MCF7 cell line, else 0 (out)BRCA - COCA: number of somatic mutations that fall inside the motif.hypermethylation_BRCA - hypermethylation_LUAD number of probes having (beta(tumor)-Beta(normal) >0.2) that fall inside the motif.Cell-lines:Hnisz: dataset is the intersection of three ChIA-PET experiments, targeting RAD21 on cell lines GM128178 and K562, and SMC1 on Jurkat cell line; junctions in the Hnisz dataset are found in at least 2 out of the 3 experiments. MCF7dataset is the breast cancer cell line. hESC dataset is the human embryonic stem cell line, junctions were inferred using the SMC1 protein-cohesin subunit as target of the ChIA-PET experiment.CTCF motifs:Using Biostrings, we identified the CTCF binding sites by targeting the 19bp long Jaspar MA0139.1 motif in the HG19 assembly, finding 107230 (104459 excluding ChrY) positions matching the motif with a score of at least 80%.Some statistics:Out of 104459 CTCF motifs,  37362 are active in some Encode ChipSeq experiments,  and 19976 are active and in boundaries in some Encode ChipSeq experiments.In MCF7 the number of boundaries is 34052 (11825 are active); in hESC the number of boundaries is 47274 (11907 are active); in Hnisz the number of boundaries is 16437 (12815 are active).4580 junctions have at least one somatic mutation for MELA (the greatest number); 15 junctions have at least one semantic mutation for COCA (the smallest number).
